# Rutin as a Potent Antioxidant: Implications for Neurodegenerative Disorders

**DOI:** 10.1155/2018/6241017

**Published:** 2018-06-27

**Authors:** Adaze Bijou Enogieru, William Haylett, Donavon Charles Hiss, Soraya Bardien, Okobi Eko Ekpo

**Affiliations:** ^1^Department of Medical Biosciences, University of the Western Cape, Robert Sobukwe Road, Private Bag X17, Bellville 7535, South Africa; ^2^Division of Molecular Biology and Human Genetics, Faculty of Medicine and Health Sciences, Stellenbosch University, Cape Town, South Africa

## Abstract

A wide range of neurodegenerative diseases (NDs), including Alzheimer's disease, Parkinson's disease, Huntington's disease, and prion diseases, share common mechanisms such as neuronal loss, apoptosis, mitochondrial dysfunction, oxidative stress, and inflammation. Intervention strategies using plant-derived bioactive compounds have been offered as a form of treatment for these debilitating conditions, as there are currently no remedies to prevent, reverse, or halt the progression of neuronal loss. Rutin, a glycoside of the flavonoid quercetin, is found in many plants and fruits, especially buckwheat, apricots, cherries, grapes, grapefruit, plums, and oranges. Pharmacological studies have reported the beneficial effects of rutin in many disease conditions, and its therapeutic potential in several models of NDs has created considerable excitement. Here, we have summarized the current knowledge on the neuroprotective mechanisms of rutin in various experimental models of NDs. The mechanisms of action reviewed in this article include reduction of proinflammatory cytokines, improved antioxidant enzyme activities, activation of the mitogen-activated protein kinase cascade, downregulation of mRNA expression of PD-linked and proapoptotic genes, upregulation of the ion transport and antiapoptotic genes, and restoration of the activities of mitochondrial complex enzymes. Taken together, these findings suggest that rutin may be a promising neuroprotective compound for the treatment of NDs.

## 1. Introduction

**Figure 1:**
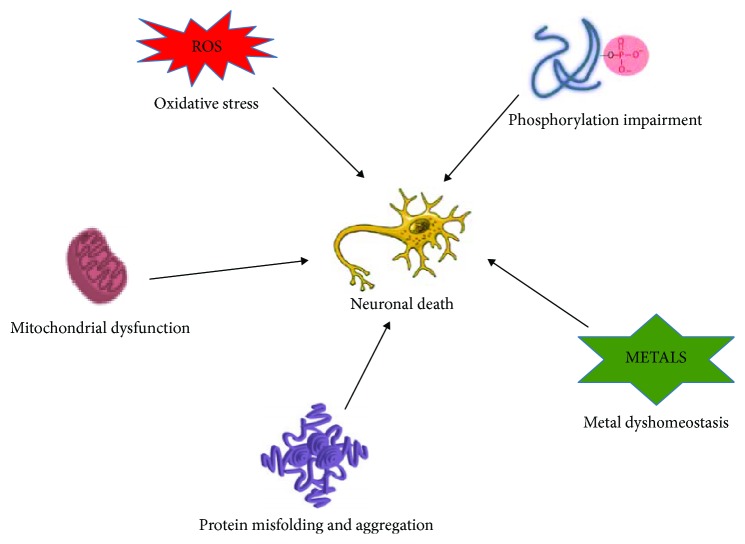
Various processes shown to be dysregulated in neurodegenerative disorders.

Neurodegenerative diseases (NDs) are regarded as an age-related group of chronic and untreatable conditions which constitutes a major threat to human health [1]. They are becoming increasingly prevalent, due to a significant increase in the size of elderly populations worldwide [2]. NDs represent the fourth highest source of disease burden in high-income countries, in terms of economic cost for society [3]. NDs are characterized by the gradual and progressive loss of neurons and diverse clinical features such as memory and cognitive impairments and others affecting a person's ability to move, speak, and breathe [4–6]. Some overlapping pathways recognized in the pathogenicity of NDs include free radical formation and oxidative stress, protein misfolding and aggregation, metal dyshomeostasis, phosphorylation impairment, and mitochondrial dysfunction [7] (Figure 1).

Oxidative stress has been shown by many studies to be a crucial player in the development and progression of NDs [8]. Oxidative stress is defined as the disturbance in balance between prooxidant and antioxidant levels and results from an imbalance between the production of reactive oxygen species (ROS) and the biological system's ability to detoxify the reactive intermediates [8]. ROS play important roles in mediating cellular activities [9, 10]; however, due to their reactivity, high amounts of ROS can cause cell death or oxidative stress [11]. While it is still unclear whether ROS is the triggering factor for NDs, they are likely to aggravate disease progression through oxidative damage and effects on mitochondria.

In view of the important roles of oxidative stress in NDs, the manipulation of ROS levels may be an encouraging treatment option to delay neurodegeneration and attenuate associated symptoms. Presently, there is no potent treatment for NDs and the available drugs are mainly focused on symptoms though with many adverse effects and limited ability to prevent disease progression [12].

Accordingly, medicinal plants such as *Hypericum perforatum* possessing antioxidant properties have been studied for their potential to attenuate neurodegenerative symptoms [13–16]. For instance, previous reports show that extracts of *H. perforatum* significantly attenuated oxidative stress by reducing lipid peroxidation [17], reducing oxidation of the mitochondrial lipid membrane [18], preserving the activities of antioxidant enzymes [19], and consequently preventing neurotoxicity in experimental models of NDs. As a result of these findings amongst others, Sánchez-Reus et al. proposed standardized extracts of *H. perforatum* as a possible treatment for elderly patients showing signs of NDs associated with elevated oxidative stress [19]. Although reports show that treatments involving *H. perforatum* are generally safe, minor adverse effects have been reported; they include dizziness, allergic reactions, restlessness, gastrointestinal symptoms, dryness of the mouth, and lethargy [20–22].

**Figure 2:**
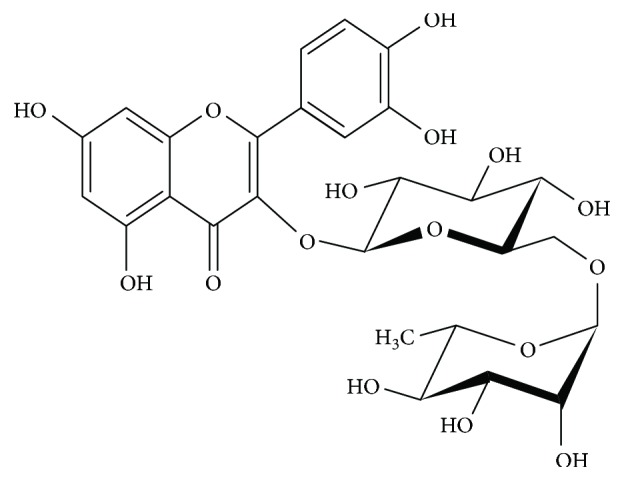
Diagram showing the chemical structure of rutin.

Similarly, there is currently an increase in the usage of natural compounds/products as potential neuroprotective agents. Examples include, curcumin, bilobalide, chitosan, and apigenin, all known to have potent protective effects on neurons [23–28]. Recently, bioflavonoids have found use in the healthcare system owing to their wide range of biological activities, low cost, and significantly high safety margins [29]. Rutin (3,3′,4′,5,7-pentahydroxyflavone-3-rhamnoglucoside, Figure 2) also called sophorin, rutoside, and quercetin-3-rutinoside is a polyphenolic bioflavonoid, largely extracted from natural sources such as oranges, lemons, grapes, limes, berries, and peaches [30, 31]. Rutin is a vital nutritional component of plants [32] and its name originates from the plant *Ruta graveolens*, which also contains rutin [33]. Chemically, it is a glycoside comprising of flavonol aglycone quercetin along with disaccharide rutinose [33]. Some studies suggest that rutin has a potential protective role in NDs due to its beneficial effects as a potent antioxidant [34, 35]. Hence, this review presents an outline of the scientific literature regarding the potential neuroprotective role of rutin in NDs.

### 1.1. Oxidative Stress and Reactive Oxygen Species

Oxygen is essential for all multicellular life but in excess, it is potentially hazardous. ROS is formed when cells exposed to oxygen continuously generate oxygen free radicals. Endogenous free radicals are generated from inflammation, mental stress, immune cell activation, excessive exercise, infection, ischemia, cancer, and aging while exogenous free radicals are produced from air and water pollution, radiation, alcohol, cooking (smoked meat, used oil, and fat), heavy or transition metals, cigarette smoke, and industrial solvents [36–38]. The main source of endogenous ROS production is the mitochondria but it can also occur in other organelles [39]. ROS include free radicals (superoxide, ^•^O^−^_2_), hydroxyl radical (^•^OH), or nonradicals (hydrogen peroxide, H_2_O_2_). ^•^O^−^_2_ is proposed to play a crucial role in ROS production and ^•^OH is recognized as the most reactive ROS that are primarily liable for the toxic effects of ROS [40].

Cellular levels of ROS may be decreased through the defence mechanisms of small-molecule antioxidants and antioxidant enzymes [41]. ^•^O^−^_2_ is reduced by superoxide dismutases (SOD) into the more stable form of H_2_O_2_. H_2_O_2_ may produce highly reactive hydroxyl radicals ^•^OH and can be reduced to H_2_O and O_2_ by catalase (CAT), glutathione peroxidase (GPx), and other peroxidases [42, 43]. The cellular antioxidant glutathione (GSH) is involved in two types of reactions. First of all, in its reduced form, GSH nonenzymatically reacts with ^•^O^−^_2_ and ^•^OH for the elimination of ROS [41, 44]. Next, GSH serves as the electron contributor for the reduction of peroxides in the GPx reaction [45]. When GSH reacts with ROS, it is oxidized (GSSG) and produces glutathione disulfide (the last product of GPx reactions). GSH can be further restored from glutathione disulfide by the reaction with glutathione reductase through a transfer of electrons from NADPH to glutathione disulfide [46]. Numerous studies have stated that GSH is involved in impeding apoptotic cell death and DNA damage in cells following oxidative stress [47, 48]. Hence, cellular antioxidants and antioxidant enzymes work together to prevent the accumulation of damaging ROS in the cell. Dysregulation of their functions is an indication of altered oxidative states, which may contribute to cell death.

The harmful effects of ROS include damage of DNA or RNA, oxidation of amino acids in proteins, oxidative deactivation of particular enzymes by oxidation of cofactors, and oxidations of polyunsaturated fatty acids in lipids (lipid peroxidation). The uninterrupted attack of protein by ROS forms protein carbonyls and nitrites, such that monitoring of their levels provides an additional measure of the effect of oxidative stress [49]. Lipid peroxidation results in the generation of lipid peroxidation products such as malondialdehyde (MDA) and thiobarbituric acid reactive substances (TBARS) [50]. Assay of TBARS measures MDA present in the sample and MDA generated from lipid hydroperoxides. An increase in free radicals is directly proportional to overproduction of MDA and is therefore a commonly used marker of oxidative stress and antioxidant status [50].

## 2. Link between Oxidative Stress and Neurodegenerative Disorders

The pathogenesis of NDs is a complex interplay between genetic and nongenetic factors [51]. Generally, nongenetic/sporadic forms represent the majority of these cases. There are a number of NDs, but for the purposes of this review, we will focus on Alzheimer's disease (AD), Parkinson's disease (PD), Huntington's disease (HD), and human prion diseases (PrDs) [1, 12].

AD is the most common ND and it primarily affects middle- to old-aged individuals, nearly one in four individuals over the age of 85 [52]. AD has various etiological factors including genetic and environmental factors [52, 53]. It is characterized by neuronal loss and atrophy in the neocortex, hippocampus, amygdala, and basal forebrain [54, 55]. Its pathophysiological hallmarks include depositions in the forms of senile plaques, extracellular *β*-amyloid (A*β*) protein, and intracellular deposits of the microtubule-linked protein tau as neurofibrillary tangles in the AD brains leading to dementia [56].

A common pathological feature in AD is the oxidation of nucleic acids, proteins, and lipids in neurons [57]. ROS interacts with polyunsaturated fatty acids in the neurons, leading to high levels of lipid peroxidation [58]. Increased levels of oxidative stress biomarkers (carbonyls, MDA, and 3-nitrotyrosine) in the blood [59, 60] and changes in the activities of antioxidant enzymes (SOD and CAT) reflect oxidative stress in the brain [61, 62].

**Figure 3:**
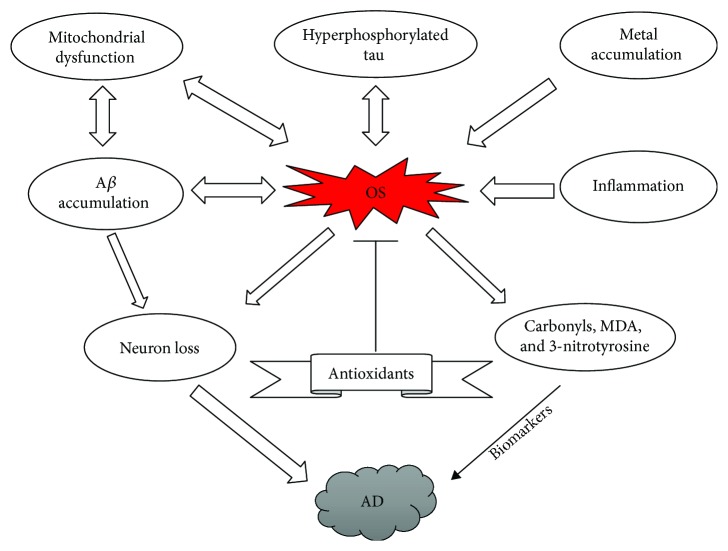
Schematic diagram showing the role of oxidative stress (OS) in Alzheimer's disease.

The underlying mechanisms (Figure 3) proposed for the initiation of oxidative stress in AD include A*β* accumulation [63, 64], hyperphosphorylated tau [65, 66], inflammation [67, 68], mitochondrial dysfunction [64, 69], and metal accumulation [70, 71].

To date, there is no treatment that can cure AD, but there are available symptomatic drug treatments consisting mostly of cholinesterase inhibitors such as donepezil, rivastigmine, and galantamine [72]. Others include memantine [73, 74], a N-methyl-D-aspartate receptor antagonist approved by the US Food and Drug Administration (FDA), and a combination of memantine with donepezil [75].

PD is characterized by chronic degeneration of dopaminergic neurons in the substantia nigra pars compacta of the midbrain [76]. This in turn results in the depletion of dopamine neurotransmitter production, which leads to motor deficits such as symptomatic rigidity, bradykinesia, postural instability, and resting tremor [77]. The cause of dopaminergic neuronal cell death in PD remains unidentified, but several factors such as oxidative stress may contribute to this degeneration and have been closely linked to other sections of neurodegenerative processes, such as *α*-synuclein, inflammation, and cell death [78–81].

Oxidative stress is believed to be a fundamental mechanism leading to cellular dysfunction in both idiopathic and familial forms of PD. An increase in protein oxidation has been detected in the substantia nigra of PD patients compared to healthy individuals [82]. Accordingly, the substantia nigra of PD patients reveals decreased levels of GSH and higher levels of oxidized proteins, DNA, and lipids [83, 84]. The accumulation of lipid peroxidation by-products has been reported in the serum and cerebral spinal fluid of PD patients while higher levels of MDA and TBARS have been reported in the substantia nigra and stratum of PD brains [85–87].

**Figure 4:**
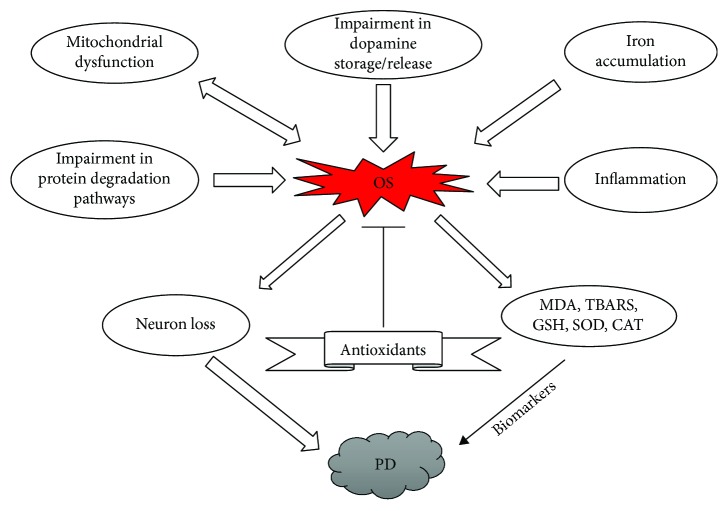
Schematic diagram showing the role of oxidative stress in Parkinson's disease.

Various mechanisms for the generation of ROS in PD include mitochondrial dysfunction, metabolism of dopamine, iron, aging, calcium, and neuroinflammation [88]. PD causing genes such as *SNCA*, *DJ-1*, *LRRK2*, *PINK1*, and *PARK2* also affect in complex ways leading to aggravation of ROS production and vulnerability to oxidative stress [88]. In addition, homeostatic processes such as mitophagy and the ubiquitin-proteasome system are affected by oxidative stress [88]. The interaction amongst these numerous mechanisms are thought to contribute to neurodegeneration in PD (Figure 4).

The primary treatment for symptomatic patients and the most effective pharmacologic agent for PD is levodopa [89, 90]. It is reported that levodopa is mostly effective at controlling rigidity and bradykinesia [89]; however, postural reflex, gait disturbance, and speech are less likely to respond. Levodopa is combined with carbidopa, because carbidopa blocks dopa decarboxylase thereby preventing peripheral conversion of levodopa to dopamine. Additionally, its combination with levodopa reduces the peripheral adverse effects of dopamine (e.g., nausea and hypotension) and increases cerebral levodopa bioavailability. Treatment with monoamine oxidase-B (MAO-B) inhibitors, amantadine (Symmetrel), or anticholinergics may modestly improve mild symptoms; nevertheless, most patients need a dopamine agonist or levodopa [91]. Furthermore, advances in brain imaging and neurosurgical techniques has highlighted surgical treatment for this disorder. In an evidence-based review, it is reported that deep brain stimulation of the subthalamic nucleus effectively improves motor function and reduces dyskinesia and motor fluctuations [90, 92].

HD is characterized by motor, cognitive, and behavioral dysfunction [93] and demonstrates an autosomal dominant mode of inheritance [94]. It is characterized by a remarkable specificity of neuronal loss and the regions most affected are the striatum, where there is usually 50–60% loss of cross-sectional area from the caudate nucleus and the putamen in advanced stages of the disease [95]. HD is linked with a triad of symptoms which includes cognitive deterioration, movement disorders, and psychiatric disturbances [95]. These signs begin subtly, most frequently between the ages of 35 to 50, but the age of onset can differ from early childhood until old age. The disease is relentlessly progressive and is deemed to be fatal 15–20 years after the onset of symptoms [95]. Classical features of HD are disturbances of motor function which include chorea (unintentional brief movements that tends to flow between body regions) and progressive deficiency of coordination of voluntary movements [95–98].

**Figure 5:**
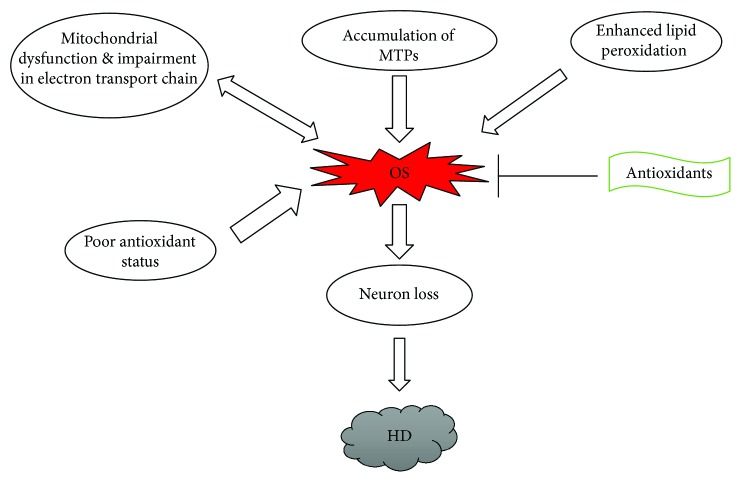
Schematic diagram showing the involvement of oxidative stress in Huntington's disease.

Convincing data supports a critical role for oxidative stress in the pathogenesis of HD [99–101] (Figure 5). Mutant huntingtin proteins (MTPs) serve as the source of ROS, owing to a substantial amount of oxidized proteins in partially purified MTP aggregates [99]. It is proposed that elevated oxidative stress is a major mechanism in the late stages of HD pathogenesis. [100]. Another mechanism involved in ROS-mediated HD pathogenesis is the impairment of the electron transport chain and mitochondrial dysfunction [102, 103]. Defects in oxidative phosphorylation have been detected in the brain tissues of HD patients [104], and enhanced lipid peroxidation accompanied by reduced GSH content has been reported in patients with severe symptoms of HD [101, 105, 106]. Substantial oxidative DNA damage has also been reported in HD mouse models [107, 108].

There are no existing treatments to alter the course of HD, but symptomatic therapies and education are effective tools used by clinicians in addressing patients and families affected by HD. Several drugs and surgical procedures have been assessed in HD for their effectiveness in subduing chorea. These include dopamine-depleting agents, agonists and antagonists, deep brain stimulation, benzodiazepines, fetal cell transplantation, acetylcholinesterase inhibitors, glutamate antagonists, antiseizure prescriptions, lithium, and cannabinoids [94, 109, 110]. Tetrabenazine is the only FDA-approved drug for HD designated for the treatment of chorea linked with HD [111, 112]. Other promising drugs shown in controlled trials to considerably lessen chorea in HD patients include amantadine [113], olanzapine [114, 115], quetiapine [116, 117], and aripiprazole [118, 119].

PrDs are related to a variety of clinical presentations and have attracted vast research awareness for many years not only due to their distinctive composition and properties but also because of their effect on public health [120–122]. Examples of PrDs include Gerstmann Sträussler-Scheinker syndrome, Creutzfeldt-Jakob disease (CJD), kuru, and fatal familial insomnia while animal PrDs include scrapie and bovine spongiform encephalopathy [123].

According to the “protein-only” hypothesis [124, 125], host-encoded cellular prion protein (PrP^C^) is converted to a different structural isoform which is known as PrP^Sc^ [120–122, 126]. It is widely regarded as the infectious agent which can duplicate itself with high conformity by enlisting endogenous PrP^C^ and that the modification between these isoforms lies strictly in its state of aggregation and its monomer conformation [120, 127]. Microscopic examination of the brains of patients with PrDs typically shows characteristic histopathologic alterations, comprising of neuronal degeneration, and vacuolation, which gives the cerebral grey matter a spongiform appearance, and a reactive increase of astroglial cells [125, 128].

**Figure 6:**
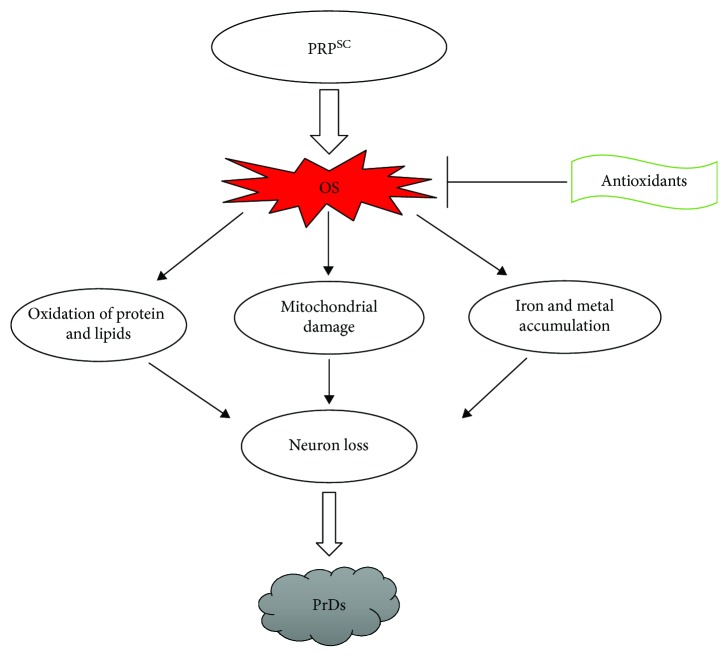
Schematic diagram showing the involvement of oxidative stress in prion diseases.

Various lines of evidence have recognized markers of oxidative stress in the brains of rodents with prion disease [129, 130] (Figure 6). Immunohistochemical studies in the brains of scrapie-infected mice have revealed the presence of lipid oxidation markers, nitrotyrosine (a marker of peroxynitrite production), and heme-oxygenase-1 (an enzyme leading to the development of antioxidant molecules), suggesting that oxidative stress might be one mechanism of neuronal loss [131, 132]. There are also indications for mitochondrial damage induced by oxidative stress in cells from brains of scrapie-infected mice and hamsters [133, 134]. Furthermore, a study by Kim et al. suggested that iron-induced oxidative stress might be a key mechanism of neuronal loss in scrapie [135].

Unfortunately, there is presently no effective treatment or disease-modifying therapy for PrDs. The search for treatments is primarily hindered by inadequate understanding of prion disease pathogenesis. However, identified drugs which show some effectiveness in treating prion diseases in *in vitro* and *in vivo* systems include quinacrine and pentosan polysulfate [136]. These compounds have been used as compassionate therapy in CJD patients; however, no therapeutic value was observed [137, 138]. Other treatment options attempted for PrDs that have had limited success include immunotherapy and vaccination [139].

## 3. General Uses of Rutin

Rutin has been shown to have an extensive array of pharmacological applications due to its numerous properties including antioxidant, anti-inflammatory, cardiovascular, neuroprotective, antidiabetic, and anticancer activities [140, 141].

Over the years, various mechanisms have been found to be responsible for its antioxidant activities in both *in vitro* and *in vivo* models. Firstly, it was reported that its chemical structure can directly scavenge ROS [142]. Secondly, it increases the production of GSH and cellular oxidative defence systems are believed to be upregulated by an increased expression of numerous antioxidant enzymes such as CAT and SOD [143–145]. Thirdly, rutin inhibits xanthine oxidase which is involved in generating ROS [146]. From the aforementioned, the optimism generated by the therapeutic potential of rutin in many health conditions in which oxidative stress is an underlying cause is understandable [34, 143, 147, 148]. The rest of this review will summarize the main findings of the neuroprotective effects of rutin in various experimental models of NDs. The various *in vitro* and *in vivo* studies are summarized in Tables 1 and 2, respectively.

### 3.1. Studies of Rutin in AD

#### 3.1.1. Toxins Used to Generate Models of AD

Several lines of evidence indicate that A*β* peptides are the key factors in AD pathogenesis [149–151]. A*β* peptide, produced from amyloid precursor protein (APP), is a very important part of amyloid plaques and has been described to be neurotoxic [152]. It is hypothesized that an anomaly in the proteolytic processing of the APP leads to an increase in the generation of A*β* peptides (such as A*β*_40–42_ and A*β*_25–35_) which in turn leads to the buildup of A*β*, a key event in the pathogenesis of AD [153, 154]. A*β* may also induce oxidative stress by causing mitochondria dysfunction which results in increased ROS and decreased levels of antioxidants such as GSH and the activity of antioxidant enzymes such as SOD, GPx, and CAT [155]. A*β*-induced ROS production is believed to aid A*β* production and accumulation, thereby quickening the progression of AD [68, 156]. Additionally, A*β* induces nitric oxide (NO) generation by upregulating the expression of nitric oxide synthase (iNOS) [157, 158] which plays a fundamental role in the series of events leading to cell death [159].

#### 3.1.2. *In Vitro* Studies

A*β* accumulation is a key feature of AD, and rutin has been shown to decrease and reverse A*β*_25–35_ fibril formation *in vitro* indicating that its action might be connected to their free radical scavenger activity and might subdue neurotoxicity [153]. Furthermore, in a different study [155], rutin acted as a multifunctional agent by inhibiting A*β* aggregation and cytotoxicity, preventing mitochondrial damage, reducing production of MDA, ROS, NO, GSSG, iNOS, and proinflammatory cytokines, and increasing CAT, SOD, GSH, and GPx levels. Yu et al. demonstrated the ability of rutin to inhibit amylin-induced neurocytotoxicity and enhance antioxidant enzyme activities in the SH-SY5Y cells [35]. Treatment of human neuroblastoma SH-SY5Y cells with rutin-loaded nanoparticles conferred protective effects on A*β*-induced cytotoxicity, decreased levels of NO, and ROS [160]. In a related activity, rutin modulated the generation of proinflammatory cytokines by reducing TNF-*α* and interleukin- (IL-) 1*β* generation in A*β*_40–42_-treated BV-2 cells [155]. Bispo da Silva et al. established that rutin treatment was not toxic to microglial cells and induced a dose-dependent increase in microglial proliferation, decreasing the mRNA levels of *TNF*, *IL-1b*, *IL-6*, and *iNOS*; reduced production of IL-6, TNF, and NO; increased production of the M2 regulatory cytokine IL-10 and arginase; and significantly inhibited the LPS-induced expression of *PTGS2*, *IL-18*, and *TGFβ* [161].

#### 3.1.3. *In Vivo* Studies

Several studies have utilized animal models as a preclinical tool to evaluate the neuroprotective potential of bioactive compounds such as edaravone and vitamin D3 in AD [162, 163]. In a study, Xu et al. [164] showed that following oral administration of rutin at a daily dose of 100 mg/kg for six weeks, rutin attenuated memory deficits in APPswe/PS1dE9 transgenic mice, reduced oligomeric A*β* level as well as downregulated microgliosis and astrocytosis, and reduced IL-1 and IL-6 levels in the brain. In an interesting and similar study by Hu et al., intravenous administration of Congo red/rutin magnetic nanoparticles (MNPs) resulted in rescue of memory deficits and amelioration of neurologic changes in the brains of APPswe/PS1dE9 transgenic mice [160]. Cheng et al. showed that rutin and exercise enhanced high-fat diet-induced cognitive defects in mice [165]. Rutin's ability to alleviate impaired cognition and memory in A*β*_25–35_-induced mouse model of AD was demonstrated by Choi et al. in 2015 [166].

Most recently, Ramalingayya et al. [167] demonstrated that pretreatment with rutin inhibited doxorubicin- (DOX-) induced ROS generation and increased DOX-induced reduction of CAT, GSH, and SOD levels in Wistar rats. Other findings include prevention of DOX-induced cell cycle and morphological changes, reduction of DOX-induced apoptosis, prevention of DOX-induced episodic-like memory deficit, prevention of rise in TNF-*α* levels, and reversal of myelosuppressive effect of DOX [167]. In a similar AD study by Ramalingayya et al. [168], rutin dose dependently improved recognition and discriminative indices in time-induced long-term as well as scopolamine-induced short-term episodic memory deficit AD models without disturbing locomotor activity. Moghbelinejad et al. demonstrated that rutin significantly increased extracellular signal-regulated protein kinase 1 (ERK1), cAMP response element-binding protein (CREB), and brain-derived neurotrophic factor (BDNF) gene expression in the hippocampus of rats. Studies show that the mitogen-activated protein kinase (MAPK) cascade that includes ERK1/2 and CREB is involved in neural plasticity and survival [169]. Long-lasting changes in synaptic plasticity and memory are the resultant effects arising from the activation the MAPK cascade [169]. BDNF affects the survival and function of neurons in the CNS and is essential for normal synaptic connection formation during growth and for learning and memory in adults [170]. They also found rutin to significantly increase memory retrieval while significantly lowering MDA levels in the hippocampus [171].

In a different type of AD model, Javed et al. showed that rutin significantly reduced intracerebroventricular streptozotocin- (ICV-STZ-) induced increase in TBARS, poly ADP-ribosyl polymerase, and nitrite in the hippocampus of rats. Rutin also significantly increased levels of GSH, GPx, glutathione reductase (GR), and CAT [172]. Furthermore, rutin also significantly improved cognitive deficits, attenuating STZ-induced inflammation by decreasing the expression of interleukin-8 (IL-8), glial fibrillary acidic protein (GFAP), cyclooxygenase-2 (COX-2), nuclear factor-*κ*B, inducible iNOS, and reduced histological abnormalities in the hippocampus [172]. In a different model of AD using zebrafish, Richetti et al. were able to show that rutin did not affect zebrafish general locomotor activity and prevented scopolamine-induced amnesia [173].

The various studies highlighted in this section demonstrates the neuroprotective capability of rutin in ameliorating the adverse effects of neurodegeneration as well as cognitive impairments associated with AD in various animal models.

### 3.2. Studies of Rutin in PD

#### 3.2.1. Toxins Used to Generate Models of PD

Over the years, neurotoxins used to induce dopaminergic neurodegeneration include 6-hydroxydopamine (6-OHDA), 1-methyl-4-phenyl-1,2,3,6-tetrahydropyridine (MPTP), 1,1-dimethyl-4,4-bipyridinium (paraquat) and rotenone [174, 175]. Seemingly, all of these toxins provoke the formation of ROS. 6-OHDA is known to be taken up by dopaminergic neurons through the dopamine transporter [174, 176]. In the neurons, oxidized molecules of 6-OHDA produces free radicals that hinders mitochondrial complex I and produces ^•^O^−^_2_ and ^•^OH which becomes toxic to dopaminergic neurons and induces microglial activation. Rotenone and MPTP are known for their ease of use in animals and their similar ability to potently inhibit complex I. After its systemic administration, MPTP swiftly crosses the blood brain barrier [175].

Once in the brain, MPTP is converted in the astrocytes by monoamine oxidase B (MAO-B) to 1-methyl-4-phenylpyridinium (MPP+) and is thereafter released into the extracellular space [175, 177, 178]. Once inside dopaminergic neurons, MPP+ accumulates in mitochondria and impairs mitochondrial respiration by impeding complex I in the electron transport chain, which induces the production of ROS [177, 179]. Rotenone is also very lipophilic and is circulated evenly throughout the brain after crossing the BBB [174, 180]. Paraquat, an herbicide, has a very close structural similarity to MPP+ and has been proposed to be a risk factor for PD [181]. A neurobehavioral syndrome characterized by reduced ambulatory activity, a decline in striatal dopamine nerve terminal density, and a significant decrease in substantia nigra dopaminergic neurons have all been associated and linked to effects from systemic administration of paraquat [182]. Experimental evidence show that paraquat crosses the BBB to cause damage to the dopamine neurons in the substantia nigra, like MPP+ [182]. In addition, sustained exposure to paraquat results in a marked accrual of *α*-synuclein-like aggregates in neurons of the substantia nigra pars compacta in mice [183].

#### 3.2.2. *In Vitro* Studies

PD has been modelled *in vitro* through the specific neurotoxic effect of the 6-OHDA on dopaminergic neurons. Neurotoxicity triggered by 6-OHDA was attenuated by rutin treatment in PC12 cells where a significant dose-dependent cytoprotective activity was detected in rutin-pretreated cells [147]. Rutin activated antioxidant enzymes including SOD, CAT, GPx, and GSH when compared to cells incubated with 6-OHDA alone in conjunction with a significantly reduced lipid peroxidation activity [147, 184]. In 2015, Magalingam et al. reported that pretreatment with rutin in PC12 cells downregulated the mRNA expression of PD-linked genes (*PARK2*, *UCHL1*, and *DJ-1*) and proapoptotic (*Casp3* and *Casp7*) genes which were upregulated in the 6-OHDA-treated PC12 cells [185]. The study showed that rutin upregulated the *TH* gene which is essential in dopamine biosynthesis and further upregulated the ion transport and antiapoptotic genes (*NSF* and *Opa1*) [185].

In a different model of PD, rutin pretreatment prevented rotenone-induced loss of SH-SY5Y cells, inhibited rotenone-induced ROS formation, and suppressed elevation of calcium [34]. Rutin attenuated rotenone-induced reduction of mitochondrial membrane potential and activation of the JNK and p38 MAPK pathways, reversed changes of Bcl-2 and Bax levels, and inhibited apoptosis and caspase-9/3 activation [34].

#### 3.2.3. *In Vivo* Studies

In one of the very few and earliest studies documenting the neuroprotective effects of rutin in *in vivo* models, oral administration of rutin significantly protected against 6-OHDA-induced increase in rotations, deficits in locomotor activity and motor coordination in male Wistar rats [78]. Immunohistochemical and histopathological findings in the substantia nigra showed that rutin protected neurons from toxic effects of 6-OHDA [78]. In a different model of PD, Sharma et al. [77] showed that rutin played an important role in attenuating behavioral, biochemical, and histological parameters after haloperidol administration in rats and further confirmed the protective effects of rutin.

These *in vivo* and *in vitro* studies exhibit the potential of rutin as a neuroprotector and suggest a role for this compound in the prevention and reversal of degenerative diseases such as PD.

### 3.3. Studies of Rutin in HD

#### 3.3.1. Toxins Used to Generate Models of HD

Animal models of HD have provided understanding into disease pathology, and previous studies of HD used toxin-induced models to study excitotoxicity-induced cell death and mitochondrial impairment, both mechanisms of HD degeneration. These models, based on quinolinic acid (QA) and 3-nitropropionic acid (3-NP), are still often used in HD studies [186]. QA is experimentally administered straight to the striatum because it is incapable of crossing the BBB [187]. Its key features include striatal neurodegeneration in rats [188, 189], mice [190], and primates [191, 192] in a strikingly similar pattern to that seen in human HD. Its advantages as a HD model includes its ease of use in more complex animals, its influences on cognitive function, numerous resemblances between pathology observed in the HD brain, and its mode of cell death that mimics the mechanism of neuronal death seen in HD brains [193–195]. 3-NP is known to irreversibly inhibit the mitochondrial enzyme succinate dehydrogenase [196, 197]. Its major advantage is that it mimics cell death seen in the HD brain through a combination of apoptosis and necrosis [186]. Instantly after administration of 3-NP, there is a surge of necrotic cell death followed by gradual apoptosis [198]. 3-NP crosses the blood-brain barrier and can be administered systemically to mice, rats, and nonhuman primates [186].

#### 3.3.2. *In Vivo* Studies

In a pioneering work on HD with rutin in 3-NP-treated rats, Suganya and Sumathi reported that oral administration of rutin (25 mg/kg and 50 mg/kg) significantly decreased protein oxidation and improved endogenous antioxidant defence system. Furthermore, rutin improved 3-NP-induced behavioral alterations and restored the activities of mitochondrial complex enzymes (I, II, IV, and V) when compared to the 3-NP-induced group [199].

In 2016, Suganya and Sumathi again reported that oral administration of rutin (25 mg/kg body weight) to Wistar rats increased the levels of nonenzymatic antioxidants (vitamin C and E) when compared to a reduction in the 3-NP-induced group. In addition, rutin protected against 3-NP-induced reduction in motor activities, muscle coordination, and activities of adenosine triphosphatases (ATPases) [200].

Most recently, Suganya and Sumathi showed that rutin restored 3-NP-induced reduction of body weight, locomotor activities, memory, and antioxidants levels. They further stated that rutin ameliorated 3-NP-induced striatal damage by reducing levels of lipid peroxides, nitrite, GFAP, and activity of acetylcholine esterase [201].

Although these few *in vivo* studies offer concrete evidence for the therapeutic potential of rutin, there exists a critical need to further elucidate and provide more evidence for the therapeutic potential of rutin in *in vitro* models of HD.

### 3.4. Studies of Rutin in PrD

#### 3.4.1. Toxins Used to Generate Models of PrD

The prion protein peptide 106–126 (PrP (106–126)) has frequently been used as a model system to study prion-induced neurodegeneration [202, 203]. This peptide induces neurotoxicity in neuronal cells owing to its amyloidogenic properties both *in vivo* and *in vitro* [204]. One of the major advantages of PrP (106–126) is that it is comparable to PrP^Sc^ in numerous respects and at the same time is more soluble and easy to deploy for cell culture experiments [205]. PrP (106–126) is rich in *β*-sheet structure, increases the membrane microviscosity of neurons and astrocytes [206], and forms aggregates that are proteinase K-resistant and detergent-insoluble [204, 207, 208]. PrP (106–126) weakens liposomes and induces liposome fusion [209].

#### 3.4.2. *In Vitro* Studies

In a pioneering study [210], the authors studied the neurotoxicity of PrP (106–126) in the HT22 hippocampal cell line and assessed the neuronal protection provided by rutin against the toxic effects of PrP (106–126). Rutin treatment blocked PrP- (106–126-) mediated increases in ROS production and NO release and delayed the decrease of neurotrophic factors that resulted from PrP accumulation. In addition, rutin mitigated PrP- (106–126-) associated mitochondrial apoptotic events by hindering mitochondrial permeability transition and caspase-3 activity and blocking expression of the apoptotic signals (Bax and PARP) in conjunction with a significantly reduced expression of the death receptor Fas and its ligand Fas-L [210].

There are currently no *in vivo* studies on the therapeutic potential of rutin in PrP models. Consequently, there is a dire need to further elucidate and provide more evidence for the therapeutic potential of rutin in more *in vitro* and *in vivo* models of PrD.

## 4. Future Perspectives and Conclusion

Numerous *in vitro* (Table 1) and *in vivo* (Table 2) studies have demonstrated the ability of rutin to ameliorate various neurodegenerative processes that trigger AD, PD, HD, and PrDs. The ability of rutin to exert its neuroprotective effects in different models of NDs could be ascribed to its antioxidant as well as antiapoptotic and anti-inflammatory activities. In addition, rutin's activation of BDNF and the MAPK cascade (ERK1/2 and CREB) signifies its involvement in plasticity and survival of neurons in the CNS.

The benchmark for authenticating rutin's neuroprotective properties is clinical trials in humans. A few clinical trials have been conducted to examine the effect of a compound from the rutin family, O-(*β*-hydroxyethyl)-rutosides (HRs) in venous disease patients with diabetes treated for a prolonged period of time [211]. HRs is obtained by substituting rutin hydroxyl groups with O-*β*-hydroxyethyl groups. Human clinical trials with rutin (in the form of HRs) have shown that it is safe and well tolerated [211]. The lack of clinical trials exploring the efficacy of rutin in NDs is of concern. This may be due to lack of sufficient data on animal models in the various NDs.

As a flavonol among similar flavonoids, rutin's low bioavailability [212] owing to high metabolism, poor absorption, and rapid excretion generally makes its prospective use as a therapeutic agent restricted. Further studies to improve its bioavailability and investigations into its protective activities in more models of NDs (most especially PrDs and motor neuron disease) would provide a solid foundation for its use in clinical trials. Rutin's ability to offer neuroprotection against pathological insult offers hope in its utilization and development as a safe and effective neurotherapeutic agent.

## Figures and Tables

**Table 1:** Summary of the protective effects of rutin in *in vitro* models of neurodegeneration.

Toxin used in cellular model	Disorder	Key findings	Reference
A*β*_25–35_-treated SH-SY5Y neuroblastoma cells and A*β*_25–35_-treated APP695-transfected SH-SY5Y (APPswe) cells	AD	↓ A*β* fibrils, ↓ *β*-secretase enzyme (BACE), ↓ ROS, ↑ GSH, ↓lipid peroxidation	[153]

A*β*42-treated SH-SY5Y and BV-2 cells	AD	↓ ROS, ↓ NO, ↓ GSSG, ↓ MDA, ↓ iNOS, ↓ MMP, ↑ GSH/GSSG ratio, ↑ SOD, CAT, and GPx, ↓ TNF-*α*, ↓ IL-1*β*	[155]

Amylin-treated SH-SY5Y neuroblastoma cells	AD	↑ cell viability, ↓ ROS, ↓ NO, ↓ GSSG, ↓ MDA and ↓ TNF-*α* and ↓ IL-1*β*, ↑ GSH/GSSG ratio, ↑ SOD, ↑ CAT, ↑ GPx, ↓ iNOS	[35]

6-OHDA-treated PC-12 cells	PD	↑ cell viability, ↑ 6-OHDA-induced reduction in SOD, CAT, GPx, and GSH, ↓ lipid peroxidation	[147]

6-OHDA-treated PC-12 cells	PD	↑ 6-OHDA-induced reduction in SOD, CAT, GPx, and GSH. ↓ lipid peroxidation, ↓ MDA	[184]

6-OHDA-treated PC-12 cells	PD	↓ Park2, ↓ UCHL1, ↓ DJ-1, ↓ Casp3, ↓ Casp7, ↑ TH, ↑ NSF, ↑ Opa1	[185]

Prion peptide-treated HT22 cells	PrD	↓ ROS, ↓ NO, ↓ apoptosis, ↓ Fas, ↓ Fas-L	[210]

6-OHDA: 6-hydroxydopamine; CAT: catalase; Fas L: Fas ligand; GPx: glutathione peroxidase; GSH: reduced glutathione; GSSG: glutathione disulfide; IL-10; interleukin 10; IL-6: interleukin 6; IL-8: interleukin 8; IL-1*β*: interleukin 1 beta; iNOS: inducible nitric oxide synthase; MDA: *malondialdehyde*; MMP: mitochondrial membrane potential; NSF: N-ethylmaleimide-sensitive factor; Opa1: optic atrophy 1; ROS: reactive oxygen species; SOD: superoxide dismutase; TH: tyrosine hydroxylase; TNF-*α*: tumor necrosis factor-*α*.

**Table 2:** Summary of the protective effects of rutin in *in vivo* models of neurodegeneration.

Toxin used in animal model	Disorder	Key findings	Reference
Doxorubicin- (DOX-) treated neuroblastoma cells (IMR32) and doxorubicin-induced cognitive dysfunction in Wistar rats	AD	↓ apoptosis, ↓ ROS, ↓ episodic memory deficit, ↓ TNF-*α*, ↑ DOX-induced reduction of catalase, GSH, and SOD	[167]

Microglial cells obtained from the cortex of Wistar newborn rats	AD	↓ TNF, ↓ IL-1b, ↓ IL-6, ↓ iNOS, ↑ IL-10, ↑ arginase, ↓ PTGS2, ↓ IL-18, ↓ *TGFβ*	[161]

^∗∗^APPswe/PS1dE9 transgenic mice	AD	↑ memory, ↑ SOD, ↑ GSH/GSSG ratio, ↓ GSSG, ↓ MDA, ↓IL-1, ↓IL-6	[164]

High-fat diet-induced obese (DIO) cognitively impaired C57BL/6J mice	AD	↓ cognitive defects	[165]

Scopolamine-treated Wistar rats	AD	↑ recognition, ↑discriminative indices	[168]

A*β*_25–35_-infused mouse model	AD	↓ impaired cognition, ↑ memory, ↓ NO, ↓ lipid peroxidation	[166]

Beta-amyloid-induced neurotoxic rats	AD	↑ ERK1, ↑ CREB, ↑ BDNF, ↑ memory retrieval, ↓ MDA	[171]

Intracerebroventricular streptozotocin- (ICV-STZ-) infused rats	AD	↓ TBARS, ↓ nitrite level, ↓ poly ADP-ribosyl polymerase, ↑ GSH, ↓ lipid peroxidation, ↓ cognitive deficits, ↓ COX-2, ↓ GFAP, ↓ IL-8, ↓ iNOS, ↓ NF-*κ*B	[172]

Scopolamine-induced zebrafish	AD	↓ amnesia	[173]

Intrastriatal injection of 6-OHDA in rats	PD	↓ 6-OHDA-induced increase in rotations, ↓ deficits in locomotor activity, ↓ motor coordination, ↑ antioxidant levels, ↑ DA, ↑ dopaminergic D2 receptors	[78]

Haloperidol-treated rats	PD	↓ catalepsy, ↓ akinesia, ↑ locomotor activity, ↑ GSH, ↑ SOD, ↓ TBARS	[77]

3-Nitropropionic (3-NP) acid-treated rats	HD	Improved 3-NP-induced behavioral alterations; restored activities of mitochondrial complex enzymes	[199]

3-Nitropropionic (3-NP) acid-treated rats	HD	Restored biochemical, behavioral, and cellular alterations	[200]

3-Nitropropionic (3-NP) acid-treated rats	HD	↑ body weight, ↑ locomotor activities, ↑ memory, ↑ antioxidant levels, ↓ lipid peroxides, ↓ nitrite, ↓ GFAP, ↓ AchE	[201]

^∗∗^Rutin loaded magnetic nanoparticles were used in this experiment; 6-OHDA: 6-hydroxydopamine; AchE: acetylcholine esterase; BDNF: brain-derived neurotrophic factor; CAT: catalase; CREB: cAMP response element binding protein; DA: dopamine; doxorubicin: DOX; ERK1: extracellular signal-regulated kinase 1; GFAP: glial fibrillary acidic protein; GPx: glutathione peroxidase; GSH: reduced glutathione; GSSG: glutathione disulfide; IL-10: interleukin 10; IL-6: interleukin 6; IL-8: interleukin 8; IL-1b: interleukin 1 beta; iNOS: inducible nitric oxide synthase; MDA: *malondialdehyde*; MMP: mitochondrial membrane potential; NF-*κ*B: nuclear factor-kappaB; NSF: N-ethylmaleimide-sensitive factor; PTGS2: prostaglandin-endoperoxide synthase 2; ROS: reactive oxygen species; SOD: superoxide dismutase; TBARS: thiobarbituric acid reactive substances; TGF*β*: transforming growth factor beta; TH: tyrosine hydroxylase; TNF-*α*: tumor necrosis factor-*α*.
